# TNF-Alpha in the Locomotor System beyond Joints: High Degree of Involvement in Myositis in a Rabbit Model

**DOI:** 10.1155/2012/637452

**Published:** 2012-03-07

**Authors:** Sture Forsgren, Lina Renström, Craig Purdam, James E. Gaida

**Affiliations:** ^1^Anatomy Section, Department of Integrative Medical Biology, Umeå University, 901 87 Umeå, Sweden; ^2^Department of Physical Therapies, Australian Institute of Sport, Belconnen, ACT 2616, Australia; ^3^School of Primary Health Care, Monash University, Frankston, VIC 3199, Australia

## Abstract

The importance of TNF-alpha in arthritis is well documented. It may be that TNF-alpha is also markedly involved in muscle inflammation (myositis). An animal model where this can be investigated is needed. A newly developed rabbit myositis model involving pronounced muscle overuse and local injections of substances having proinflammatory effects was therefore used in the present study. The aim was to investigate the patterns of TNF-alpha expression in the developing myositis and to evaluate the usefulness of this myositis model for further TNF-alpha research. Human rheumatoid arthritis (RA) synovial tissue was examined as a reference. TNF-alpha immunoexpression and TNF-alpha mRNA, visualized via in situ hybridization, were detected in cells in the inflammatory infiltrates of the affected muscle (soleus muscle). Coexistence of TNF-alpha and CD68 immunoreactions was noted, suggesting that the TNF-alpha reactive cells are macrophages. Expression of TNF-alpha mRNA was also noted in muscle fibers and blood vessel walls in areas with inflammation. These findings demonstrate that TNF-alpha is highly involved in the myositis process. The model can be used in further studies evaluating the importance of TNF-alpha in developing myositis.

## 1. Introduction

Tumour necrosis factor alpha (TNF-alpha) is one of the most frequently studied pro-inflammatory cytokines. It drives the activation and recruitment of inflammatory cells, amplifies the production of other pro-inflammatory cytokines, and activates nuclear transcription factors, thereby promoting and maintaining the inflammatory response [1]. TNF-alpha is likely to be a key cytokine in several autoimmune diseases such as rheumatoid arthritis (RA), inflammatory bowel disease, systemic sclerosis, and systemic lupus erythematosus [1–3].

 A very large number of studies have been performed investigating the importance of TNF-alpha in arthritis, especially RA. TNF-alpha has also attracted interest in recent years for its possible role in skeletal muscle damage. Increased protein degradation, as well as decreased body weight and food consumption, was demonstrated when TNF-alpha was administered to rats via a catheter into the external jugular vein [4]. Crush injury in mice leads to elevated TNF-alpha levels in skeletal muscle tissue [5] and there is an increase in TNF-alpha serum levels in response to repetitive strain injuries [6]. However, the experiments that have been performed have sometimes yielded apparently conflicting results. For example, an experiment in which TNF-alpha was administered to mice via an osmotic pump led to accumulation of inflammatory cells in skeletal muscles but no signs of atrophy or injury [7]. Furthermore, the results of studies on TNF receptor knockout and TNF-alpha antibody-neutralized mice indicate that TNF-alpha can actually be involved in the recovery of muscle function after traumatic muscle injury [8]. Therefore, it might be that the role of TNF-alpha in muscle injury varies with the type, severity, and stage of the injury [9]. In humans, TNF-alpha is known to be intimately involved in cachexia [10], a complex condition characterised by progressive muscle loss that affects up to 13% of patients with RA [11]. Nevertheless, a recent study showed acute elevation of TNF-alpha not to affect markers of systemic or skeletal muscle turnover in healthy humans [12].

Remarkably little data is available on the role of TNF-alpha in situations where there is a pronounced infiltration of inflammatory cells in the muscle tissue, that is, myositis. From studies in tissue other than muscle, it is known that macrophages and other immunoactive cells such as monocytes, mast cells, and neutrophils are responsible for TNF-alpha production [13–16]. Data addressing a possible TNF-alpha production by inflammatory cells in myositis comes almost entirely from studies of patients affected by a group of diseases known as “idiopathic inflammatory myopathies” (inflammatory myopathies) [17, 18]. These autoimmune diseases include mainly the subgroups inflammatory myopathic polymyositis, dermatomyositis, and inclusion body myositis [19]. In these conditions, inflammatory cell-related TNF-alpha expression is localised predominantly to macrophages [18]. TNF-alpha is also expressed in the inflammatory cells in crush-injured and transplanted muscle autografts in mice [7]. Finally, blockade of TNF-alpha in the dystrophic (mdx) mouse, which is the most frequently used model of Duchenne's muscular dystrophy, reduces TNF-mediated adverse responses to exercise-induced muscle damage [20, 21]. However, without further information, it is difficult to reach conclusions on the importance of TNF-alpha and the possible usefulness of TNF-blocking in muscle disorders, including in myositis [22]. Furthermore, it should be stressed that the majority of information on the TNF system for skeletal muscle tissue has come from studies of cultured myoblasts (e.g., [23]). Animal models are needed to advance our understanding of the disease mechanisms of TNF-alpha that are involved in myositis.

Our laboratory has developed a rabbit model of marked muscle (m. triceps surae) and tendon overuse that, when combined with injections of substances eliciting pro-inflammatory effects, results in significant myositis [24]. This model causes myositis that morphologically resembles that seen in inflammatory myopathies [17, 18] but without having an apparent autoimmune origin. The types of white blood cells involved in the inflammatory infiltrates were defined [24]. The model leads to a muscle pathology that to some extent resembles the morphology seen in overuse musculoskeletal disorders (see [25], for a review, see [26]). In studies using this model, we noted evidence of local glutamate signaling in the cells of the inflammatory infiltrates within the muscle tissue [24]. We have taken advantage of this model in order to examine the TNF-system during myositis development. Thus, the aim of this study was to examine the pattern of TNF-alpha expression in one segment of the triceps surae muscle (the soleus muscle) affected by myositis using immunohistochemistry and in situ hybridization in order to get an insight into the possible usefulness of this model for further studies on the importance of TNF-alpha in myositis.

## 2. Material and Methods

### 2.1. Animals and Experimental Procedures

#### 2.1.1. Animals

Twenty-eight adult female New Zealand white rabbits were used for the studies. The animals had an average weight of 4 kg and ranged in age from 6 to 9 months. The animals were kept in ordinary cages allowing good freedom of movement.

 Six of the animals corresponded to control nonexercised animals (subgroup 1) and 22 were assigned to an exercise protocol leading to marked overuse of the triceps surae muscle (subgroups 2–5). In order to increase the muscle affection, including the degree of inflammation, the muscle overuse was combined with paratendinous injection treatment (cf. the following). As a control for this, five of the exercised animals (subgroup 2) were given control substance (NaCl) just outside the tendon of the triceps surae muscle (i.e., the Achilles tendon). In essence, these five animals did not develop myositis (Song et al., unpublished observations). For the purpose of achieving muscle inflammation, 17 of the exercised animals (subgroups 3–5) were in parallel to being subjected to marked overuse, given local injections of pro-inflammatory substance (substance P and/or endopeptidase inhibitors; Captopril, DL-Thiorphan) outside the Achilles tendon. Substance P was given as this neuropeptide has well-known pro-inflammatory effects [27] and the injections of the endopeptidase inhibitors were given in order to diminish endopeptidase activities and thereby lead to more pronounced effects of substance P.

For clarification of all the various animal groups, see Table 1.

#### 2.1.2. Exercise Procedure

The animals were exposed to an exercise procedure designed to cause marked overuse of the triceps surae muscle and the associated tendon (the Achilles tendon). The procedure is performed according to previously described procedures [28], with some modifications [29]. Throughout the experiment, the rabbits were kept under anaesthesia, induced by intramuscular (i.m.) injections of diazepam (5 mg/mL; 0.2 mL/kg) and fentanylfluanison (0.2-0.3 mL/kg). Fentanylfluanison (0.1 mL/kg) was injected each 30–45 min during the experiment in order to maintain the anaesthesia. Each experimental session lasted for 2 hours. For analgesia, buprenorphine (0.03 mg/kg) was given subcutaneously (s.c.) after each experiment session. The experiment was repeated every second day for one week (4 exercise sessions in total).

 An apparatus (kicking machine) was used to achieve passive repetitive flexions and extensions of the right ankle joint; a pneumatic piston attached to the right foot produced the movements. During the plantar flexion, an active contraction was furthermore induced by electrical stimulation via surface electrodes (Pediatric electrode 40 426 A, Hewlett Packard, Andover, MA, USA) placed 2 cm apart over the triceps surae muscle of the right leg. The stimulation was synchronized with the plantar flexion movement of the piston by a microswitch, which trigged the stimulator unit (Disa stimulator Type 14E10, Disa Elektronik A/S, Herlev, Denmark). An impulse of 0.2 ms duration was delivered 85 ms after the initiation of the plantar flexion at an amplitude of 35–50 V. The movement frequency was 150 repetitions per minute. The left leg was not attached to the kicking machine. The pelvis was strapped down and there were no ankle movements on the left side. One day after the final exercise session, the animals were sacrificed by an overdose of Pentobarbital. For further details about the apparatus and the exercise protocol, see [24, 28–30].

#### 2.1.3. Injection Treatments

Injections were given into the loose connective tissue around the Achilles tendon, that is, in the paratenon region. The injections were given directly after each of the 2-hour exercise periods. The substances injected were (a) NaCl (0.91% w/v, volume: 1 mL) (subgroup 2; *n* = 5), (b) Substance P (10^-8^ *μ*mol/mL) and Captopril (Sigma) (c4042, 30 *μ*mol/kg) both in distilled water (volume: 1 mL) and Dl-Thiorphan (N-[(RS)-2-Benzyl-3-mercaptopropanoyl]-glycine) (Sigma) (500 *μ*g/mL; 0.02 mL) (subgroup 3; *n* = 5), (c) Captopril (Sigma) (c4042, 30 *μ*mol/kg, dissolved in distilled water, volume 1 mL) + DL-Thiorphan (Sigma) (500 *μ*g/mL, 0.02 mL) (subgroup 4; *n* = 6), and (d) Captopril (Sigma) alone (c4042, 30 *μ*mol/kg, dissolved in distilled water, volume 1 mL) (subgroup 5; *n* = 6). For further details, see Table 1.

#### 2.1.4. Grouping of the Animals

Based on recent observations in our group (Song et al., unpublished observations) and as recently reported [24], it has become obvious that subgroups 3–5 develop myositis. Therefore, these subgroups were grouped together and further on referred to as the “myositis group”. As there are minimal or no signs of myositis in the NaCl-treated subgroup (subgroup 2), the animals in this group were grouped together with the nonexercised animals (subgroup 1), comprising the “non-myositis group”.

#### 2.1.5. Collection of Muscle Samples: Sectioning

**Muscle Samples**

After the animals were sacrificed, the right triceps surae muscle was dissected out and immediately transported on ice to the laboratory. Samples conforming to the soleus muscle part (5–8 × 10 mm) were dissected out and fixed by immersion overnight at 4°C in an ice-cold solution of 4% formaldehyde in 0.1 M phosphate buffer (pH 7.0). The samples were thereafter thoroughly washed in Tyrode's solution containing 10% sucrose at 4°C overnight, mounted on thin cardboard in OCT embedding medium (Miles Laboratories, Naperville, Ill, USA), frozen in propane chilled with liquid nitrogen, and stored at −80°C. Series of 5 *μ*m thick sections were cut using a cryostat. The sections were mounted on slides precoated with chrome-alum gelatine and were then processed for immunohistochemistry. Other sections were processed for morphology or in situ hybridization.

#### 2.1.6. Human RA Tissue Studied in Parallel

As a reference, human RA synovial tissue was analyzed. The tissue was fixed and further processed in the same way as were the rabbit specimens (cf. the following).

### 2.2. Processing for Immunohistochemistry and Morphology

#### 2.2.1. Staining for Demonstration of Morphology

One section from all specimens was stained in Harris Haematoxylin solution for 2 min. These sections were then rinsed in distilled water, dipped in 0.1% acetic acid for a few seconds, and then washed in running water. Counterstaining was achieved by immersion in eosin for 1 min. The sections were dehydrated in ethanol and mounted in Permount.

#### 2.2.2. Immunohistochemistry

Sections of all specimens were processed for immunohistochemistry. The sections were pretreated with acid potassium for 2 min, a procedure found to enhance specific immunofluorescence reactions [31]. Thereafter followed incubation for 20 min in a 1% solution of Triton X-100 (Kebo lab, Stockholm) in 0.01 M phosphate buffer saline (PBS), pH 7.2, containing 0.1% sodium azide as preservative, and three 5 min washes in PBS. The sections were then incubated for 15 min in 5% normal donkey serum (code no: 017-000-121, Jackson Immune Research Lab. Inc.) in PBS. Next, incubation with the primary antibody, diluted in PBS (pH 7.4), occurred in a humid environment for 60 min at 37°C. After incubation with specific antiserum, and three 5 min washes in PBS, another 15 min incubation in normal donkey serum followed. Next, the sections were incubated with either of these donkey antigoat IgGs for 30 min at 37°: FITC-(fluorescein isothiocyanate-) conjugated AffiniPure donkey antigoat IgG (Jackson ImmunoResearch Lab Inc, dilution 1 : 100) or Alexa FluorO 488 donkey antigoat (Invitrogen, dilution 1 : 300). The sections were thereafter washed in PBS and then mounted in Vectashield Mounting Medium (H-1000) (Vector Laboratories, Burlingame, CA, USA). Examination was carried out in a Zeiss Axioscope 2 plus microscope equipped with epifluorescence optics and an Olympus DP70 digital camera.

#### 2.2.3. Double Stainings

To clarify the TNF-alpha immunoreaction pattern in relation to that of white blood cells, double stainings were made. As it is frequently emphasized that macrophages [13, 16] show TNF-alpha expression, double stainings for TNF-alpha/macrophage marker (CD68) were performed. Double stainings for TNF-alpha/T-cell-neutrophil marker were also performed.

 Alexa FluorO donkey antigoat was used as secondary antibody for TNF-alpha immunolabelling, and TRITC (tetramethylrhodamine isothiocyanate-) conjugated rabbit antimouse antibody was used for stainings for CD68 and T-cell/neutrophil marker. For detailed information about the staining procedures for TNF-alpha, see above. When doing double stainings for CD68 and T-cell/neutrophil marker, 5% normal rabbit serum (code no: X0902, DAKO Cytomation, Glostrup, Denmark), diluted in 0.1% BSA (bovine serum albumin) in PBS, was used as normal serum, and TRITC-conjugated rabbit antimouse antibody (R0276, DAKO Cytomation), diluted 1 : 40 in 0.1% BSA in PBS, as the secondary antibody.

#### 2.2.4. TNF-Alpha Antibody and Control Stainings

An antibody against TNF-alpha produced in goats was used (AF-210-NA; R&D Systems). Various dilutions were trialled to achieve the optimal fluorescence to background ratio, with a dilution of 1 : 50 found to be optimal. The supplier reports that this antibody is directed against *E. coli*-derived recombinant human TNF-alpha. The TNF-alpha-specific IgG was purified by human TNF-alpha affinity chromatography. It is described to be specific via having the ability to neutralize the biological activity of recombinant human TNF-alpha. Of note, the TNF-alpha amino acid sequence homology between species is reported to be highly conserved and TNF-alpha DNA sequence comparison shows an overall high sequence homology between various species (including rabbit) [32].

 In control stainings, preabsorption of the primary antibody with TNF-alpha antigen (T6674; Sigma; 20 *μ*g/mL antiserum) was performed overnight at 4°C. Control staining also included staining when the primary antibody was omitted.

#### 2.2.5. Antibodies against CD68 and T-Cell/Neutrophil Marker and Reference Concerning Double Stainings

A macrophage (CD68) antibody (M0814) from DAKO Cytomation (Glostrup, Denmark) was used. It is an affinity purified mouse monoclonal antibody and was used at a dilution of 1 : 100 in 0.1% BSA in PBS. The antigen for this antibody is glycosylated transmembrane glycoprotein, which is mainly located in lysosomes. A mouse antirabbit T-cell and neutrophil antibody (MCA805G) from AbD Serotec (Oxford, UK) was furthermore used. It is an affinity purified mouse monoclonal antibody against a cell surface antigen, which is expressed by a subset of T-cells, thymocytes, neutrophils, and platelets in rabbits. The dilution was 1 : 100 in 0.1% BSA in PBS.

 Stainings performed in a parallel project on rabbit soleus muscle [24] were used as a reference (control) for the current double stainings. In that project, double stainings were performed using the same CD68 and T-cell/neutrophil marker antibodies as in the current double stainings. In this previous study, double stainings were made against an antibody produced in goats (against VGluT2; Santa Cruz), that is, being of the same type as the TNF-alpha antibody used in the current study. In these reference stainings, the same types of secondary antibodies as described previously were utilized.

## 3. Processing for In Situ Hybridization

In situ hybridization was used as a complementary method to detect the expression of TNF-alpha, namely, at the mRNA level. A digoxigenin-(DIG) hyperlabeled oligonucleotide probe (ssDNA) for detection of rabbit TNF-alpha mRNA was used on sections from myositis (4 specimens) and nonmyositis (1 specimen) groups (GD1001-DS custom designed; GeneDetect, New Zealand). The antisense sequence of the probe was CGGCGAAGCGGCTGACAGTGTGAGTGAGGAGCACGTAGGAGCGGCAGC. The procedures were performed according to an established protocol [33], using an alkaline phosphatase-labeled anti-DIG antibody for detection [34]. The probe for TNF-alpha mRNA was used at 50 ng in 15 *μ*L of hybridization solution.

 The tissue specimens were cut into 10 *μ*m thick fresh cryosections using a cryostat (with a knife washed in 70% EtOH in DEPC [diethylpyrocarbonate]-H_2_O) and mounted onto Super Frost Plus slides (nr.041200, Menzel Gläser). The protocol that thereafter followed was that previously used in our laboratory for detection of mRNA for other substances (e.g., [34–36]).

 An alkaline phosphatase-(AP) labelled anti-DIG antibody (Roche, Germany, 11 093 274 910) was used for detection. The sections were finally mounted in Pertex mounting medium.

The corresponding sense DIG-hyperlabeled ssDNA probe was used as a negative control. As a positive control probe, a *β*-actin antisense probe (GD5000-OP) was used, comparisons being made with sense *β*-actin probe (GeneDetect, New Zealand).

**Ethics**

The study protocol was approved by the local ethical committee at Umeå University.

## 4. Results

### 4.1. Morphology

**Figure 1:**
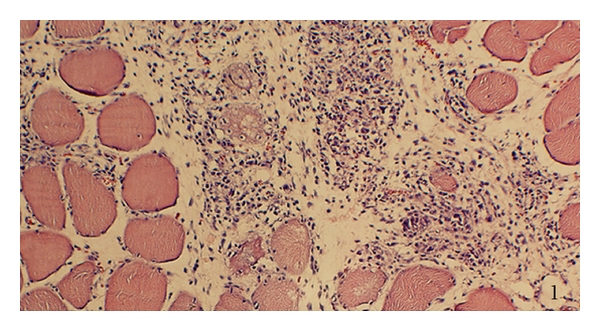
Soleus muscle of a specimen of the myositis group in a section stained with htx eosin. There is a marked inflammatory infiltrate (middle part): myofibers to the left and to the right.

Myositis was observed in subgroups 3–5, and these are now collectively being referred to as the “myositis group” (cf. above). The most noteworthy feature was the presence of an inflammatory infiltrate (Figure 1), although muscle fiber changes were also observed, including muscle fiber necrosis (cf. [24]). Variations in the levels of myositis were observed between the different subgroups, as well as between different animals within the subgroups. The inflammatory infiltrates were seen in some parts of the specimens. In the other groups (subgroups 1 and 2), there were no or very marginal changes seen and these are now collectively referred to as the “nonmyositis group”.

### 4.2. Reference Studies Concerning the TNF-Alpha Antiserum Used

It was considered relevant to examine a reference tissue regarding the demonstration of TNF-alpha. Human RA synovial tissue was therefore analysed to provide reference information for the particular TNF-alpha antibody used, as it a well-known fact that there is a marked TNF-alpha expression in the inflammatory infiltrates in the synovial tissue of patients with RA [37].

**Figure 2:**
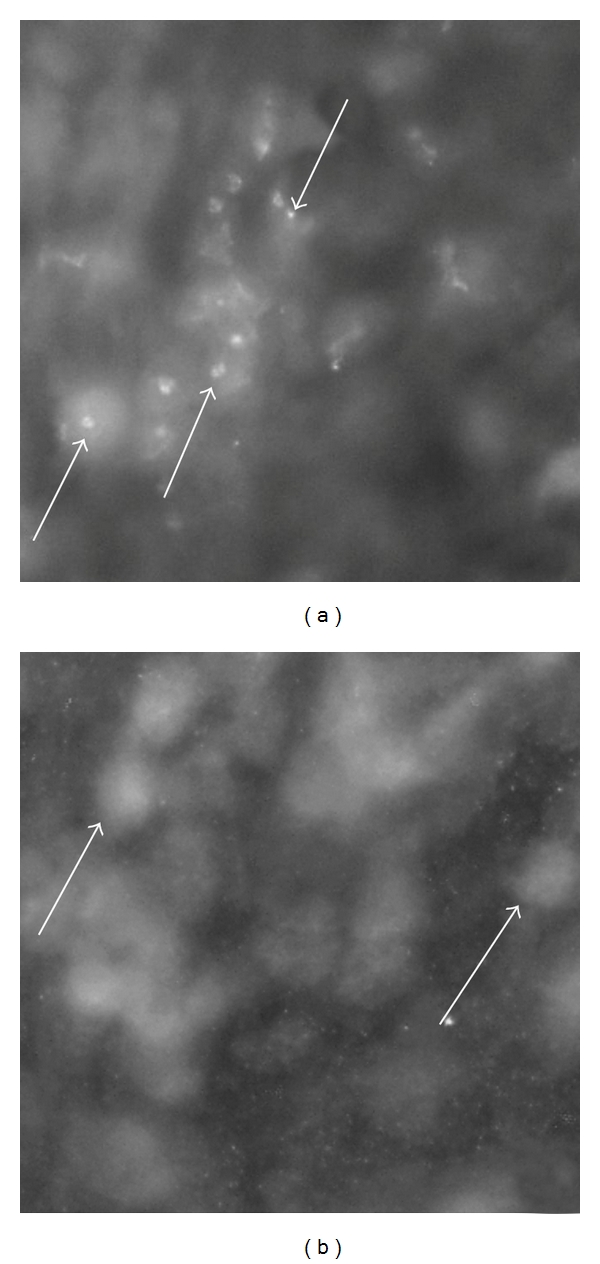
(a, b) Sections of synovial tissue of a patient with rheumatoid arthritis. The sections were stained for demonstration of TNF-alpha (a) and for TNF-alpha after preabsorption with TNF-alpha antigen (b). Inflammatory cells show specific immunoreaction in (a) (arrows). There are no specific immunoreactions in these in (b) (arrows). The inflammatory cells had in parallel sections been identified via staining for routine morphology (htx eosin).

**Figure 3:**
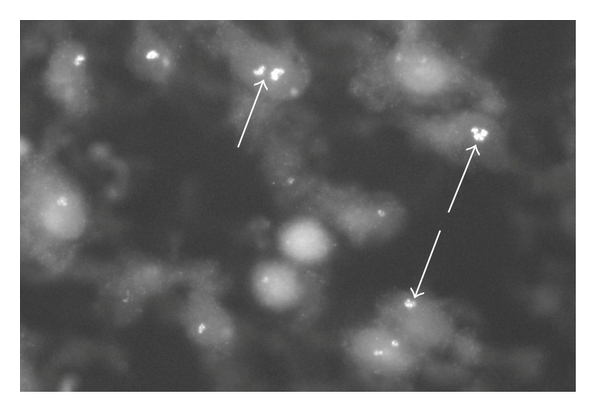
Staining for demonstration of TNF-alpha in a synovial tissue specimen of a rheumatoid arthritis patient. There are specific reactions in inflammatory cells (arrows). The reactions show a granular appearance.

 We observed that mononuclear-like cells of the human synovial tissue exhibited immunoreactions when incubated with the TNF-alpha antiserum (Figures 2 and 3). The reactions were in high magnification seen in the form of intracellular granular reactions (cf. Figures 2 and 3). The specificity of the reactions was confirmed via preabsorption with synthetic antigen (Figure 2). The cells occurred as parts of immune cell aggregates or as isolated cells in the synovial tissue.

### 4.3. TNF Alpha Immunoreactions in the Rabbit Soleus Muscle

**Figure 4:**
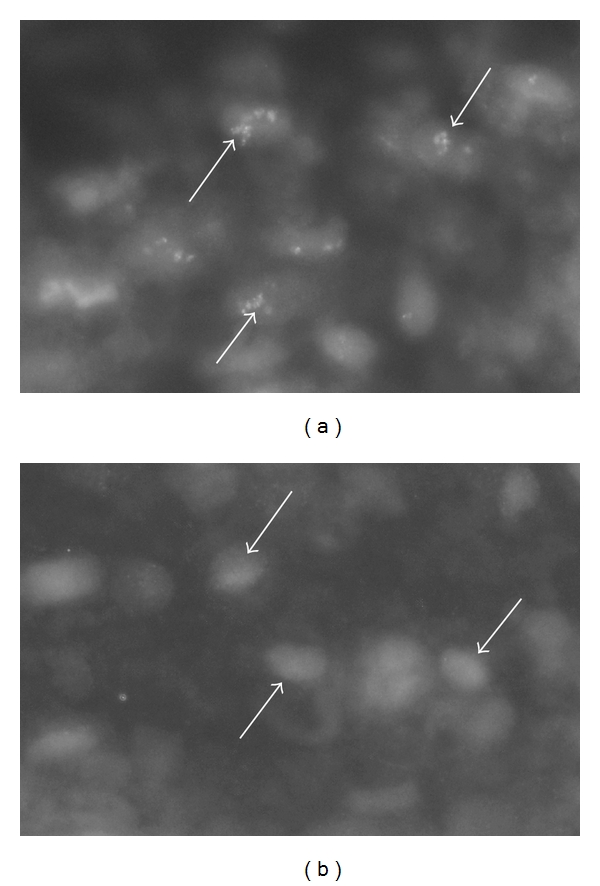
Sections of a myositis specimen stained for TNF-alpha (a) and for TNF-alpha after preabsorption with TNF-alpha antigen (b). The cells of an inflammatory infiltrate show immunoreactions for TNF-alpha (a) (arrows). There are no specific reactions in (b) (arrows at some of the cells).

**Figure 5:**
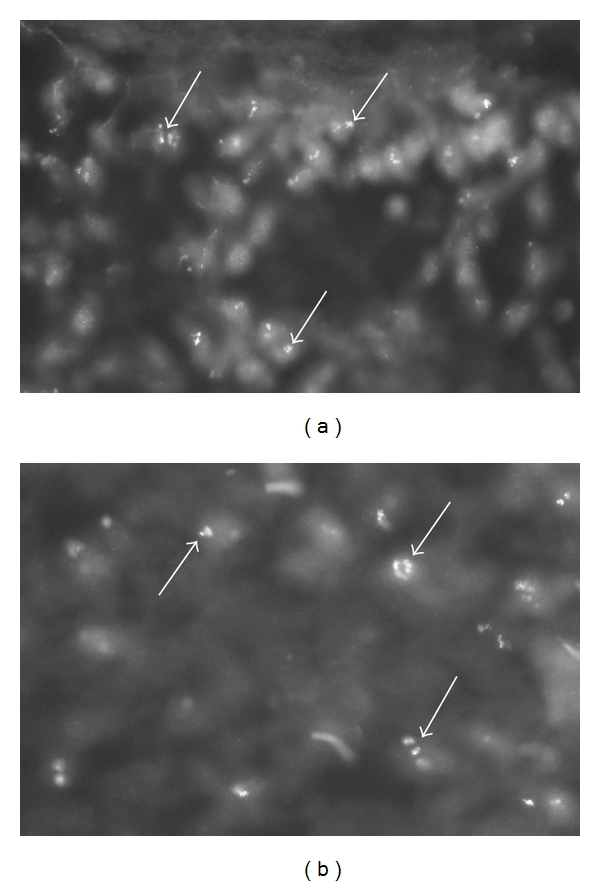
(a, b) Sections of myositis specimens after processing for TNF-alpha. Cells show specific immunoreactions (arrows). The reactions show a granular appearance.

**Figure 6:**
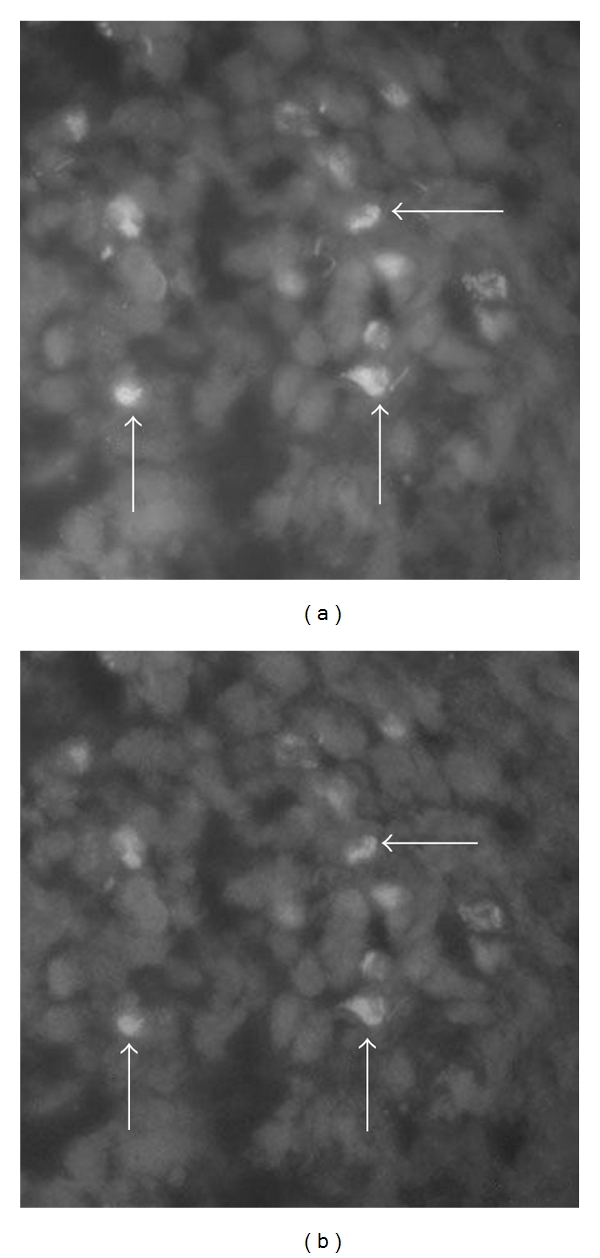
(a, b) Inflammatory infiltrate in a soleus muscle (myositis specimen). Double-staining for demonstration of TNF-alpha (a) and CD68 (b). Immunoreactions are seen in the same cells (arrows).

Pronounced TNF-alpha immunoreactions were observed in cells of the inflammatory infiltrates (Figures 4 and 5) in all subgroups in the myositis group. It was noteworthy that the immunoreaction patterns seen in the cells resembled those observed for the mononuclear-like cells of the human RA synovial tissue. Thus, the reactions showed a granular pattern in high magnification (Figures 4 and 5). In lower magnification, the reactions were of a more diffuse type (Figure 6(a)). The specificity of the reactions was verified via preabsorptions (Figure 4).

 Fibroblasts in the connective tissue did also to some extent display TNF-alpha immunoreactions, however the reactions were very faint. No specific TNF-alpha reactions were noted for blood vessel walls, the nerve fascicles, muscle spindles, and the muscle fibers (not shown).

### 4.4. Results of Double Stainings

In order to clarify the patterns of cellular reactions for TNF-alpha in the inflammatory infiltrates, double stainings for TNF-alpha/CD-68 and TNF-alpha/T cells and neutrophil marker were performed. It was found that CD68 coexisted with TNFalpha in cells in the inflammatory infiltrates (Figure 6). On the other hand, colocalization between TNF-alpha and T cells and neutrophil marker was not observed (not shown). In the reference studies (cf.  Section 2) using the same secondary antisera and the same white blood cell markers but a primary goat antibody not directed against TNF-alpha, completely different colocalization patterns were noted [24].

### 4.5. In Situ Hybridization

**Figure 7:**
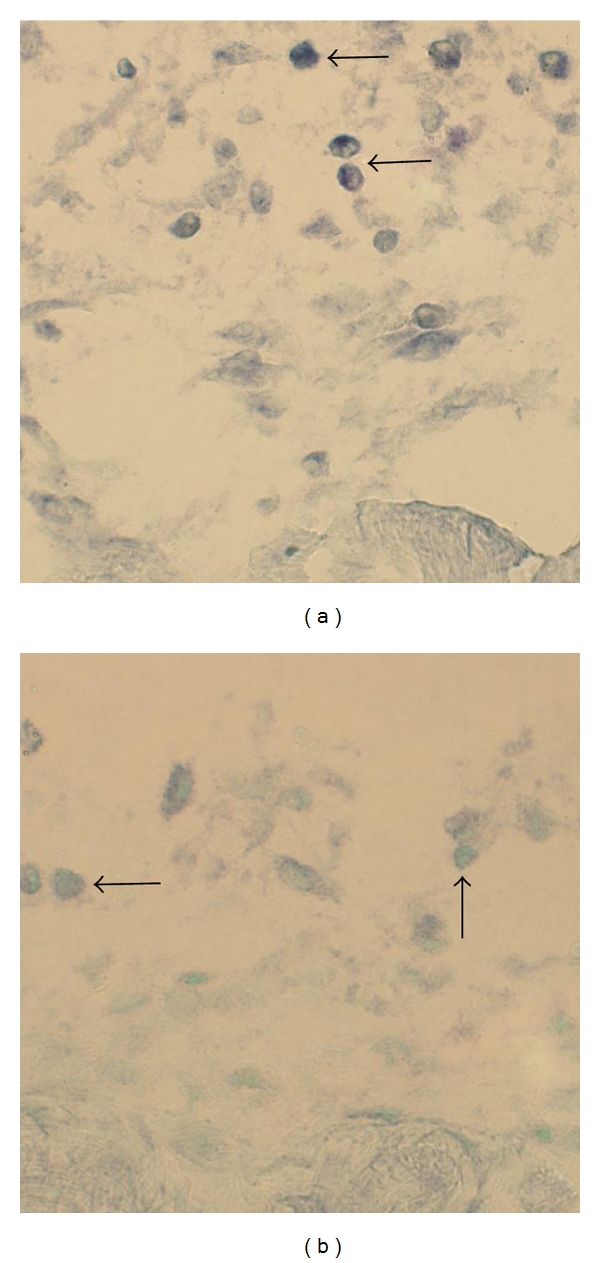
In situ hybridization for the demonstration of TNF-alpha mRNA in inflammatory cells. Adjacent sections are shown: antisense staining (a), and sense staining (b). There are reactions in (a) but not in (b). The arrows point at inflammatory cells.

**Figure 8:**
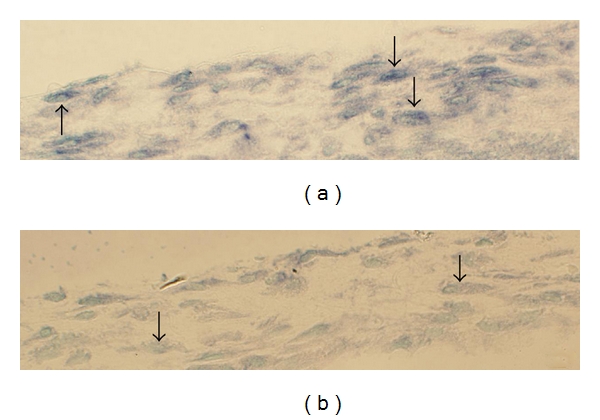
In situ hybridization for the demonstration of TNF-alpha mRNA in fibroblasts. Adjacent sections are shown: antisense staining (a), and sense staining (b). There are reactions in (a) but not in (b). The arrows point at fibroblasts.

**Figure 9:**
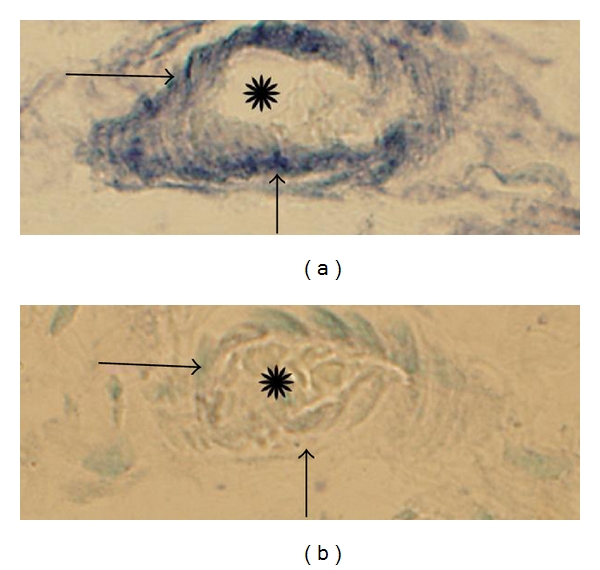
In situ hybridization for the demonstration of TNF-alpha mRNA in a small blood vessel: antisense staining (a), and sense staining (b). The vessel was located in the proximity of an inflammatory infiltrate. Arrows point at the wall. There are reactions in the wall in (a) but not in (b). Asterisks are in the lumen.

**Figure 10:**
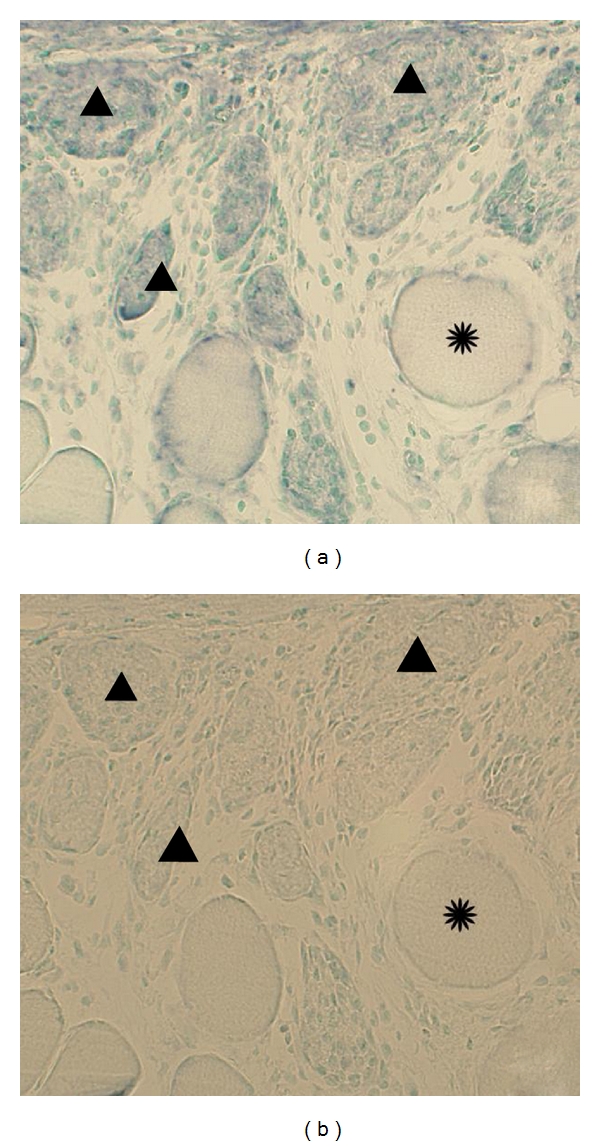
In situ hybridization for the demonstration of TNF-alpha mRNA in the muscle tissue. Adjacent sections are shown, one processed with antisense probe (a), the other with sense probe (b). There are reactions in muscle fibers in (a) but not in (b). Arrowheads indicate corresponding muscle fibers in (a) and (b). As verified via examinations of parallel sections processed for morphology, the reactive muscle fibers are infiltrated by inflammatory cells. Certain muscle fibers appear to be unaffected (asterisk).

Reactions for TNF-alpha mRNA were revealed for white blood cells of the inflammatory infiltrates in the myositis specimens (Figure 7). Reactions were also seen for fibroblasts (Figure 8) and sometimes for blood vessel walls (Figure 9) and muscle fibers (Figure 10). The muscle fibers and blood vessels for which reactions were seen were located in the regions with inflammatory infiltrates. It was noted that the muscle fibers with reactions for TNF-alpha mRNA were often infiltrated by inflammatory cells. The majority of the muscle fibers and blood vessels in the tissue of the myositis samples had no demonstrable reaction. There were no reactions at all in the musculature and the blood vessel walls in the nonmyositis samples. No reactions were noted for nerve fascicles and muscle spindles in any of the specimens from the myositis or non-myositis groups.

## 5. Discussion

It is well known that TNF-alpha is highly involved in arthritis, notably in RA. Accordingly, in our reference studies in the present investigation we found that TNF-alpha was expressed in mononuclear-like cells in the RA synovial tissue. Detection of TNF-alpha reactions was thus clarified from the methodological point of view, and verifications were obtained via preabsorption stainings. With this as a basis, studies on TNF-alpha in myositis were performed.

A unique model for the production of myositis in rabbit musculature (the soleus muscle) was utilized. The main finding was that cells in the inflammatory infiltrates in the myositis muscles were found to express TNF-alpha at both at the mRNA and protein levels. Colocalization between TNF-alpha and CD68 was noted for these cells. Expression of TNF-alpha in macrophages has previously been noted in other situations (e.g., [13, 16]), including inflammatory myopathies [17]. In contrast, in our recent studies using the current myositis model, expression of the vesicular glutamate transporter VGluT2 was noted in white blood cells in the inflammatory infiltrates other than macrophages [24]. A further main finding was that the muscle fibers and blood vessel walls in areas showing inflammatory infiltration exhibited TNF-alpha mRNA and that fibroblasts also were seen to exhibit TNF-alpha mRNA.

From a methodological point of view, it was clear that muscle fibers, blood vessel walls, and fibroblasts exhibited TNF-alpha mRNA but that no reactivity (muscle fibers, blood vessel walls) or very weak reactivity (fibroblasts) was noted at the protein level. The production level in these locations is therefore likely to be low, which precluded clear detection with our immunohistochemical methods. It is also possible that our in situ hybridization method detects very small quantities of TNF-alpha mRNA. Nevertheless, it has previously been shown that TNF-alpha can be expressed not only in inflammatory cells but also in injured muscle fibers and fibroblasts in response to muscle injury (crush-injury) [7] as well as in muscle fibers and cells in the connective tissue in inflammatory myopathies [17, 18].

The patterns of morphologic appearances of the inflammatory infiltrates and other morphologic changes seen resembled the appearances that can be seen in the muscle tissue in inflammatory myopathies [38]. Nevertheless, preliminary analysis using ELISA detecting anti-Jo-1 antibodies, which are known to correlate with disease activity for patients with inflammatory myopathy [39], does not lend proof to the theory that the myositis in our model is autoimmune in origin (unpublished observations). However, further studies on this aspect are warranted.

As noted previously, TNF-alpha immunoreactions in inflammatory cells invading muscles affected by myositis have previously only been documented in biopsies from patients with inflammatory myopathies [17, 18] and in muscle of mice in response to crush-injury [7]. Thus, in combination with this previous work, our results imply that TNF-alpha is intimately involved in the inflammatory process in myositis. Indeed, it has been suggested that TNF-alpha may have a role in the pathogenesis of the myositis in the inflammatory myopathies [40, 41] and that a marked inflammatory response involving TNF-alpha may be directly responsible for damaging muscle fibres in myopathic conditions [42].

Whether or not the TNF-alpha produced by the cells of the inflammatory infiltrates is entirely responsible for pro-inflammatory and damaging effects remains open to speculation. It is well known that TNF-alpha administration can have pro-inflammatory and detrimental effects, for example, leading to various catabolic changes as seen in studies on cultured skeletal muscle cells [4, 43, 44]. However, there is a marked discrepancy in the literature regarding the effect of TNF-alpha on the musculature. Some studies on myoblast cell culture show that TNF-alpha administration does not have catabolic effects (e.g., [45]), and other studies documenting accumulations of inflammatory cells in skeletal muscle in response to TNF-alpha administration [4] show no decrease in skeletal muscle proteins and no signs of muscle atrophy or injury. Perhaps these discrepancies reflect a dual role of TNF-alpha where in some circumstances inflammatory cell derived TNF-alpha can play a protective role [46] and also be involved in the recovery of muscle function after traumatic injury [9] and in muscle regeneration [47]. The discrepancies may also reflect the fact that different methods have been used in the studies that have been performed. The results in preliminary studies on inflammatory myopathies suggest that TNF blocking might be useful [48], but it is also emphasized that further studies are needed in order to clarify if this type of treatment is indeed useful [22]. Results of in vitro studies suggest that targeting TNF-alpha might be worthwhile in myositis [49, 50] and studies on dystrophic mdx mice subjected to wheel exercise indicate that TNF blockade can reduce myofiber necrosis [20, 21]. The use of anti-TNF treatment in studies on a rat model of repetitive reaching and grasping leads to an improvement in grip strength and attenuated task-induced increases in inflammatory cytokines, including TNF-alpha [51].

Although the use of other animal models have shown inflammation in muscle tissue in response to various forms of exercise [50, 52], the myositis model used in the current experiment is clearly distinguishable from these models. Thus, in contrast to these models, it leads to a marked presence of inflammatory infiltrates in the muscle tissue, that is, a morphology resembling that seen in inflammatory myopathies. In fact, no experimental myositis model exists which resembles the one used here and in which a marked presence of inflammatory infiltrates becomes present in the muscle tissue. Those for which such an infiltration has been demonstrated are the model of crush-injury described above [7] and models designed to help understand the mechanisms of inflammatory myopathies that occur in man. In these latter cases, myositis is induced by various infectious agents [53, 54], immunization with muscle components, for example, myosin [55, 56], and intraperitoneal injections with lipopolysaccharide [57]. The TNF system has not been examined in any of these myositis models replicating the inflammatory myopathies seen in man. Interestingly, there is evidence indicating a relationship between the inflammatory myopathies and another condition, muscular dystrophy, in the form of complex interactions between immunological and nonimmunological features of the diseases [58].

A noteworthy aspect with the currently used model is that marked overuse is applied in the procedures. Nevertheless, a limitation of the present study is that the relative contributions of the exercise protocol and the injections of the proinflammatory substances to the observed myositis are unclear. Forthcoming studies will clarify this issue. In any case, the model will, as it is currently used, provide the opportunity to evaluate the effects of interference with TNF-alpha actions in myositis development.

## 6. Conclusions

 An animal model in which the importance of TNF-alpha for myositis development can be followed has previously been lacking. Using a newly established rabbit model of myositis development, a marked TNF-alpha expression has here been shown for the cells of the inflammatory infiltrates within damaged muscle. There was thus a clear evidence of local TNF-alpha production via infiltrated inflammatory cells, presumably leading to secondary inflammation-modifying effects. Using in situ hybridization, it was also seen that TNF-alpha mRNA was detected for muscle fibers and blood vessel walls in regions of inflammatory infiltrates. The current model can be used for further studies on the importance of TNF-alpha in the development of myositis and to document the expression patterns of other cytokines and signal substances in this condition.

## Figures and Tables

**Table 1:** Summary of the five subgroups of animals analyzed. The number of animals for which soleus specimens were analysed is shown. Subgroups 3–5 had been given pro-inflammatory substances/endopeptidase inhibitors in combination with exercise. The subgroups 1 and 2 are collectively referred to as comprising the “nonmyositis group” and those of subgroups 3–5 as comprising the “myositis group” in the text.

Subgroup	Exercise	Injection	Number of animals
1	No	—	6
2	Yes	NaCl	5
3	Yes	Substance PCaptoprilDL-Thiorphan	5
4	Yes	CaptoprilDL-Thiorphan	6
5	Yes	Captopril	6
